# Real-Time fast PCR amplification using designated and conventional real time thermal cycler systems: COVID-19 perspective

**DOI:** 10.1371/journal.pone.0276464

**Published:** 2022-10-20

**Authors:** Md. Walid Hossain, Mohabbat Hossain, Khalid Arafath, Subarna Sayed Ety, Md. Mahade Hasan Shetu, Mazbahul Kabir, Farjana Akther Noor, Kaiissar Mannoor

**Affiliations:** 1 Molecular Biology Laboratory, OMC Healthcare (Pvt.) Limited, Rupnagar, Dhaka, Bangladesh; 2 Infectious Disease Laboratory, Institute for Developing Science & Health Initiatives, Dhaka, Bangladesh; Waseda University: Waseda Daigaku, JAPAN

## Abstract

The study aimed to shorten multiplex RT-PCR run time for detection of SARS CoV-2 N1 and N2 sequences and human RNase P (RP) sequence as internal mRNA control using conventional and designated real time thermal cycler systems. Optimization of Fast PCR protocol using plasmid-based N1 and N2 positive control and synthetic version of human RP was done on Applied Biosystems (ABI) QuantStudio^TM^5 (conventional), ABI 7500 Fast Dx (designated), and CFX96 Touch Real Time Detection System, Bio-Rad (conventional). Finally, a performance evaluation of Fast PCR was performed in terms of sensitivity, specificity, and precision. For a 40-cycle PCR with optimized Fast PCR protocols on QuantStudio^TM^5, ABI 7500 Fast Dx, and CFX96 Touch (conventional), standard/regular versus Fast PCR run times (min) were 84 vs. 49, 96 vs. 48, and 103 vs. 61, thereby saving 35, 48, and 43 min, respectively. For each thermal cycler, Standard and Fast PCR generated identical shapes of fluorescence curves, Ct values, and (3) R^2^ (0.95 to 0.99) for 5 10-log dilution panels of each positive control. The fast PCR approach generated results with 100% sensitivity and specificity. Median test comparisons between standard PCR and Fast PCR Cts of COVID-19 samples did not produce significance (p>0.5), suggesting that Fast PCR and Standard PCR were comparable. Also, the median and mean of each target had closely-related values, further suggesting that the two approaches were comparable. That is, there is an equivalency between Conventional and Fast PCR instruments for detection of COVID-19.

## Introduction

After the first report on the detection of SARS-CoV-2 on 8 March 2020 in Bangladesh, there was an alarmingly gradual increase in the rate of COVID-19 infections [1]. Even technologically developed countries had been facing an unexpectedly difficult situation to cope with the analysis of a huge number of specimens from suspected COVID-19 patients [2]. During that time real time RT-PCR was the only acceptable method for the detection of COVID-19 infections [3]. Bangladesh was poorly prepared to handle such an emergency. In the beginning, there were very few molecular laboratories equipped with real time RT-PCR facilities for COVID-19 testing. Sometimes a service-providing laboratory was receiving double or triple or even quadruple the numbers of specimens beyond the capacity of analysis. As a result, some patients had to wait a couple of days to get the test results. In the meantime, many more COVID-19 test laboratories had been established across the country. Despite such development, laboratories could not cope in an analysing huge number of samples during the deadliest second wave of COVID-19 circulation in the country [4]. It is assumed that same precarious situation happened in other countries [5].

Although use of real time PCR has been decreasing over time for COVID-19 detection due to SARS-CoV-2 vaccination coverage, rapid antigen testing, and serological analysis, it still remains as the gold standard for detection of hundreds of pathogens in the diagnostic and research laboratories and in times of epidemic and pandemic emergency [6, 7].

Typical run time for real time PCR is between 1.5 hours to 2 hours. Recently, many life science industries including Qiagen, ThermoFisher, and New England Biolabs have developed Fast PCR kits. Fast cycling PCR buffers/master mix with super-efficient Hot Start DNA Polymerase facilitate rapid amplification of primer-specific PCR products with significantly reduced run time on existing conventional real time thermal cycler systems. In addition, many industries are developing and/or are upgrading their real time thermal cycler systems for Fast PCR, such as ABI 7500 Fast Dx, MBS NEXTGEN PCR for ultra-fast cycling, and MIC PCR of Bio Molecular Systems etc. [8].

We previously reported a highly sensitive in-house multiplex real time RT-PCR using laboratory-based master mix for detection of SARS-CoV-2 [9]. We asked whether we could use our master mix for detection of SARS CoV-2 using previously described primers and probes for Fast amplification of SARS-CoV-2 N1 and N2 target sequences and human RNase P (P) target as internal control using designated (ABI 7500 Fast Dx Real-Time PCR System) and conventional thermal cycler systems (on QuantStudio™5 System and CFX96 Touch Real-Time Detection System). Thus, the aim of the study was two-fold including (1) optimization of Fast PCR protocol and comparison of the optimized Fast PCR results with standard/regular PCR results for SARS-CoV-2 N1 and N2 target sequences and human RNase P target using ABI7500 Fast Dx, and CFX96 Touch Real-Time Detection System, and (2) performance evaluation of Fast PCR protocol for detection of SARS-CoV-2 using nasopharyngeal swab specimens of suspected COVID-19 patients, and sensitivity and specificity of the Fast PCR approach for detection of COVID-19. Among these PCR systems, ABI 7500 Fast Dx is a designated Fast PCR cycler, although it can be used for regular amplification. The ultimate goal of the study was to significantly shorten the PCR run time.

## Methods

### Study design and sample collection

This retrospective study was designed and conducted at Molecular Biology Laboratory of OMC Healthcare (Pvt.) Ltd., Dhaka, Bangladesh. The validation part of the study was performed at institute for Developing Science and Health initiatives (ideSHi). The nasopharyngeal swab samples (NPS) were collected by trained medical technologists as per SARS-CoV-2 NPS collection guideline. The samples were collected in a 3 ml viral transport media (VTM) and transported to ideSHi COVID-19 laboratory through proper implementation of cold chain. Since retrospective samples were used, verbal consents over phone calls were taken. The study was approved by the ethics committee of Institutional Review Board of CRO which performed the validation study.

### SARS-CoV-2 positive controls for optimization of Fast PCR protocols

Plasmid positive controls containing known copies of 2019-nCoV_N as target gene and synthetic version of human RP gene known as Hs_RPP30 were purchased from IDT and were used as positive control and internal control, respectively. 2019-nCoV_N gene plasmid positive control was used as a target for both N1 and N2 sequences.

### Sample information for performance evaluation of the Fast PCR approach

Frozen samples including 60 COVID-19 positive and 60 COVID-19 negative nasopharyngeal swab specimens (NPS) in VTM, which were previously confirmed by Sansure Novel Coronavirus (2019-nCoV) Nucleic Acid Diagnostic Kit (Sansure Biotech Inc., China) were used for validation and precision studies.

### Viral RNA extraction from clinical specimens

The sample tubes containing nasopharyngeal swab specimens in VTM were opened in a biosafety class-II type A2 cabinet (Model: LA2-4A1-E. Esco) and viral nucleic acid was extracted using Quick-RNA^TM^ Viral Kit (Zymo Research, U.S.A) following manufacturer’s protocol.

### Primer and probes for SARS-CoV-2 detection

We received USA CDC primers and probes from IDT for COVID-19 detection. The primers were designed to target highly conserved N1 and N2 sequences of SARS-CoV-2 N gene. Human RNase P (RP)-specific primers and probes were used for detection of internal control. The primer and probe sequences for detection of N1 and N2 sequences of SARS-CoV-2 and human RP (RNase P) sequences were as follows. 2019-nCoV_N1 Forward Primer: 5’- GAC CCC AAA ATC AGC GAA AT-3’, 2019-nCoV_N1 Reverse Primer: 5’-TTC TGG TTA CTG CCA GTT GAA TCT GA-3’, 2019-nCoV_N1 Probe: 5’-/6-FAM/ACC CCG CAT TAC GTT TGG TGG ACC /3BHQ_1/-3’, 2019-nCoV_N2 Forward Primer: 5’-GAT TAC AAA CAT TGG CCG CAA ATT GC-3’, 2019-nCoV_N2 Reverse Primer: 5’-TAG CGC GAC ATT CCG AAG AAC G-3’, 2019-nCoV_N2 Probe: 5’-/VIC/ACA ATT TGC CCC CAG CGC TTC AG/3BHQ_1/-3’, RNase P Forward Primer: 5’-AGA TTT GGA CCT GCG AGC G -3’, RNase P Reverse Primer: 5’-GAG CGG CTG TCT CCA CAA GT-3’, RNAse P Probe: 5’-/CY5/TTC TGA CCT GAA GGC TCT GCG CG /3BHQ_1/-3’.

### Selection of RT-PCR kit for validation study

The Reverse Transcription Real-Time Polymerase Chain Reaction (RT-PCR) was performed on 60 COVID-19 positive and 60 COVID-19 negative samples using RealDetect^TM^ COVID-19 RT-PCR Kit (Cat# M02-06-73) which is similar to Sansure Novel Coronavirus (2019-nCoV) Nucleic Acid Diagnostic Kit (CE-IVD, NMPA, FDA-EUA) (Sansure Biotech Inc., China) in terms of sensitivity and specificity.

These COVID-19 positive and negative samples were initially selected by RT-PCR amplification using Sansure Novel Coronavirus (2019-nCoV) Nucleic Acid Diagnostic Kit (Sansure Biotech Inc.).

### PCR instruments and thermal cycle profiles for standard and Fast PCR approaches

For qualitative detection of SARS-CoV-2 using both regular and Fast approaches, 3 different real time PCR systems including QuantStudio™5, Applied Biosystems™ 7500 Fast Dx, and CFX96 Touch Real-Time Detection System were used. A PCR reaction volume of 20μl containing 10μl Mastermix, 1.5μl Primer & Probe solution mix, and 8.5μl extracted RNA sample from a suspected SAR-CoV-2 patient was run on each of the mentioned thermal cyclers. Thermal cycling profile and ramp rate were optimized for each PCR system and have been elaborated as follows.

**Table:** 

Thermal cyclers	QuantStudio™5 Real-Time PCR System		Applied Biosystems™ 7500 Fast Dx		CFX96 Touch Real-Time Detection System		No. of Cycles
RAMP rate	2.5°C/sec		Fast mode (100%)		5°C/sec		
Steps	Temp. (°C)	Time	Temp. (°C)	Time	Temp. (°C)	Time	
RT Steps	55	5 min	55	8 min	55	8 min	1
	95	1 min	95	1 min	95	1 min	
PCR Steps	95	7 sec	95	5 sec	95	5 sec	40
	60	30 sec	60	30 sec	60	30 sec	

### Evaluation of sensitivity of Fast amplification PCR

Nasopharyngeal swab specimens (NPS) from suspected COVID-19 patients were analyzed using Sansure Novel Coronavirus (2019-nCoV) Nucleic Acid Diagnostic Kit (Sansure Biotech Inc., China) for selection of 60 COVID-19 positive and 60 COVID-19 negative samples. To determine sensitivity of the Fast PCR approach, RNAs from COVID-19 positive and COVID-19 negative were run for both standard and Fast PCR.

### Evaluation of specificity of Fast amplification PCR

To investigate whether N1 and N2 primer pairs targeting the N gene of SARS-CoV-2 could cross-react with other closely-related coronaviruses, such as SARS-CoV and MERS-CoV, plasmids containing N gene of SARS-CoV and MERS-CoV (Integrated DNA Technology, USA) in addition the plasmid containing 2019-nCoV_N gene were used.

### Performance evaluation of Fast PCR approach as per precision analysis using Ct values

Precision analysis was performed using mean±SD and medians of Ct values of N1, N2, and RP of COVID-19 positive specimens and RP internal control, respectively.

### Statistical analysis

For method validation, mean±SD of Ct value of each target upon repeated measurements was calculated. Linear regression analysis using Ct values across sample dilution panels of positive controls was performed and R^2^ values were compared between standard and Fast PCR. For performance evaluation in terms of Ct values of COVID-19 positive samples, median test was performed and Ct values of each target were compared between standard PCR and Fast PCR (AtoZmath.com). Comparisons were also made between mean and medians of standard and Fast PCR. A *P* value <0.05 was considered statistically significant.

## Results

Comparison of run time and time saved between Standard RT-PCR and optimized Fast RT-PCR protocols for QuantStudio™5 Real-Time PCR System, Applied Biosystems™ 7500 Fast Dx Real-Time PCR System, and CFX96 Touch Real-Time PCR detection System.

The objective of this part of the study was to save time by application of Fast amplification (RT)-PCR for detection of COVID-19. Table 1 shows a comparison between standard and optimized Fast amplification real time RT-PCR protocol in terms of RT time, RT enzyme inactivation time, and PCR stage time including polymerase activation time, PCR product denaturation time, and combined annealing and extension time for the mentioned thermal cycler systems. For standard/regular real time RT-PCR, reverse transcription on QuantStudio™5 Real-Time PCR System, Applied Biosystems™ 7500 Fast Dx Real-Time PCR System and CFX96 Touch Real-Time PCR detection System took 10 min, 10 min, and 8 min, respectively, whereas RT on QuantStudio™5 Real-Time PCR System and Applied Biosystems™ 7500 Fast Dx Real-Time PCR System and 8 min and 5 min, respectively and there was no change of RT time for CFX96 Touch Real-Time PCR detection System. On the other hand, post-RT holding times for RT enzyme inactivation before standard PCR starts were 8 min, 1 min, and 1 min for QuantStudio™5 Real-Time PCR System, Applied Biosystems™ 7500 Fast Dx Real-Time PCR System and CFX96 Touch Real-Time PCR Detection Systems respectively, whereas post-RT enzyme inactivation before Fast PCR starts took 1 min for all thermal cycler systems.

**Table 1:** Comparison of run time and time saved between standard PCR and optimized Fast RT-PCR protocols.

Protocol	Stages	Temperature and time		
		QuantStudio™ 5 Real-Time PCR System	Applied Biosystems™ 7500 Fast Dx Real-Time PCR System	CFX96 Touch Real-Time PCR detection System
Standard PCR	Reverse transcription	55°C- 10 minutes	55°C- 10 minutes	55°C- 10 minutes
	RT enzyme inactivation	95°C- 1 minute	95°C- 1 minute	95°C- 1 minute
	Polymerase activation & Cycle denaturation	95°C- 10 seconds	95°C- 10 seconds	95°C- 10 seconds
	Combined annealing and extension	60°C- 1 minute	60°C- 1 minute	60°C- 1 minute
Fast PCR	Reverse transcription	55°C- 5 minutes	55°C- 8 minutes	55°C- 8 minutes
	RT enzyme inactivation	95°C- 1 minute	95°C- 1 minute	95°C- 1 minute
	Polymerase activation & Cycle denaturation	95°C- 7 seconds	95°C- 5 seconds	95°C- 5 seconds
	Combined annealing and extension	60°C- 30 seconds	60°C- 30 seconds	60°C- 30 seconds
Standard RT-PCR run time		86	95	103
Fast RT-PCR run time		49	48	61
Time saved		37	47	42

Fast RT-PCR protocol has been optimized so that it gives results which are similar to Standard RT-PCR result in terms of shape of fluorescence intensity curves, PCR efficiency and precision. The protocols have been optimized on QuantStudio™ 5 Real-Time PCR System, Applied Biosystems™ 7500 Fast Dx Real-Time PCR System, CFX96 Touch Real-Time PCR detection System. In addition to the protocols, the last three rows of Table 1 shows total RT-PCR run time for Standard and Fast PCR across the thermal cyclers and time saved (last row) due to Fast PCR amplification.

For standard PCR, cycle denaturation times on QuantStudio™5 Real-Time PCR System, Applied Biosystems™ 7500 Fast Dx Real-Time PCR System and CFX96 Touch Real-Time PCR detection Systems were 10 sec, 15 sec, and 10 sec respectively, whereas for Fast PCR, cycle denaturation times were 7 sec, 5 sec, and 5 sec, respectively. On the other hand, combined annealing and extension time for standard vs. Fast PCR on QuantStudio™5 Real-Time PCR System, Applied Biosystems™ 7500 Fast Dx Real-Time PCR System and CFX96 Touch Real-Time PCR detection Systems were 45 sec vs. 30 sec were 1 min vs. 30 sec, 45 sec vs. 30 sec and 30 sec/30 sec, respectively.

To complete the PCR of 40 cycles on QuantStudio™5 Real-Time PCR System, Applied Biosystems™ 7500 Fast Dx Real-Time PCR System, and CFX96 Touch Real-Time PCR detection Systems, standard versus Fast PCR run times were 86 min vs. 49 min, 95 min vs. 48 min, and 103 min vs. 61 min, thereby saving 37 min, 47 min, and 42 min, respectively. The findings together indicate that Fast PCR amplification could save significant amounts of time compared to standard PCR. It should be informed here that Applied Biosystems™ 7500 Fast Dx Real-Time PCR System which is designated for Fast PCR, although it can be operated for Standard PCR also. On the other hand, QuantStudio™5 Real-Time PCR System and CFX96 Touch Real-Time PCR detection System are conventional real time thermal cycler systems.

Fast RT-PCR protocol has been optimized so that it gives results which are similar to Standard RT-PCR result in terms of shape of fluorescence intensity curves, PCR efficiency and precision. The protocols have been optimized on QuantStudio™ 5 Real-Time PCR System, Applied Biosystems™ 7500 Fast Dx Real-Time PCR System, CFX96 Touch Real-Time PCR detection System. In addition to the protocols, the last three rows of Table 1 shows total RT-PCR run time for Standard and Fast PCR across the thermal cyclers and time saved (last row) due to Fast PCR amplification.

### Protocol validation for optimization of Fast PCR

The validation criteria which were considered for optimization of the Fast PCR protocols for different thermal cycler systems included (1) shapes of the fluorescence curves, (2) Ct values of known copy number positive controls, namely N1 and N2 targets and RP internal control, and (3) R^2^ values generated through linear regression analysis.

**Fig 1:**
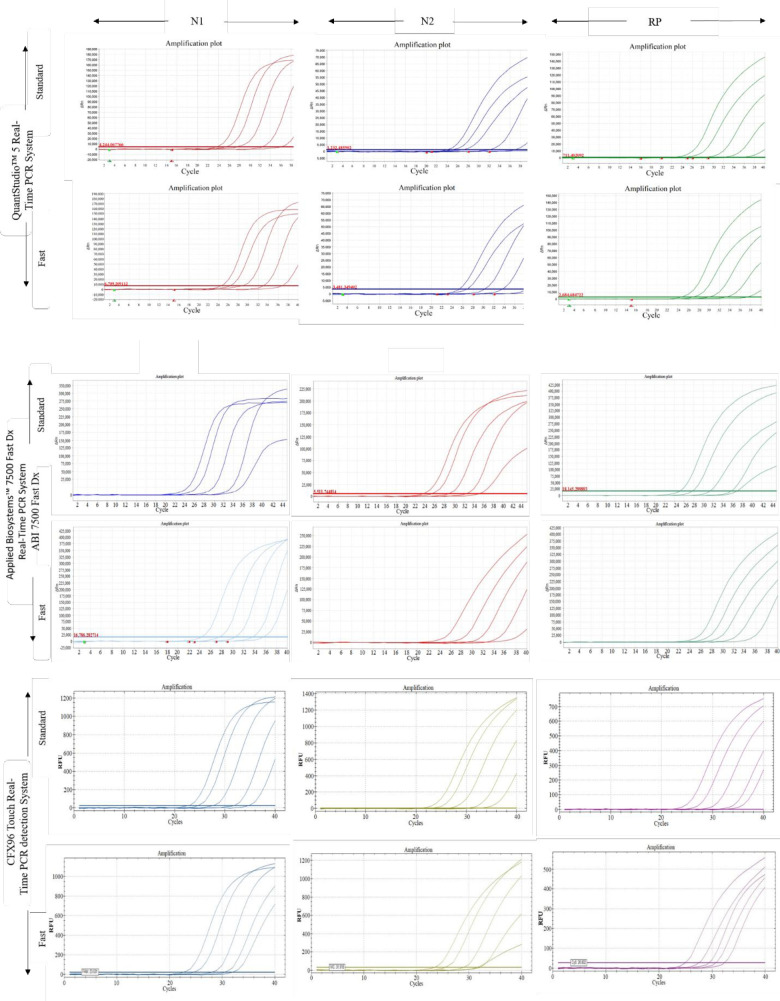
Fluorescence intensity curves of SARS CoV-2 targets, namely N1 (FAM) and N2 (VIC), and human RNase P (CY5) due to amplification using standard PCR and Fast PCR protocols.

First, we wanted to check the qualities of fluorescence intensity curves for FAM, VIC, and CY5 to represent degrees of amplification of N1, N2, and RP targets, respectively by standard and Fast PCR approaches for different thermal cycler systems. For all the panels in **Fig 1**, Fast PCR amplification curves were typical in terms of shapes and comparable to standard PCR curves. Each panel shows distinct separation of fluorescence curves for the dilution panel of each target.

This figure shows limit of detection (LOD) showing fluorescence intensity curves of 10000,1000,100,10 and 1 copy of N1, N2 and RP targets for standard and fast PCR approaches across three different thermal cycler systems including QuantStudio^TM^5 Real Time PCR System, Applied Biosystems™ 7500 Fast Dx Real-Time PCR System, and CFX96 Touch Real-Time Detection System. The upper two rows of panels indicate results for QuantStudio^TM^5 Real-Time PCR System. The 3rd and 4th rows of panel indicate results for Applied Biosystems™ 7500 Fast Dx Real-Time PCR System, whereas the lower 2 rows of panels indicate results for CFX96 Touch Real-Time Detection System.

The Ct values of N1/FAM, N2/VIC, and RP/CY5 have been shown as mean±SD across dilution panels of positive control for standard and Fast PCR (Table 2). When PCR amplification was started on QuantStudio™5 Real-Time PCR System with 10000 copies of N1, the Ct values due to Standard and Fast PCR amplification were 23.62±0.07 and 23.58±0.13 respectively, followed by 26.23±0.64 and 26.58±0.26, 29.52±0.82 and 28.96±0.52, 31.96±0.25 and 31.28±0.40, and 35.32±0.24 and 35.35±0.58 for N1 copy numbers of 1000, 100, 10, and 01, respectively. Similarly, the Ct values of N2 due to Standard and Fast PCR amplification of 10000, 1000, 100, 10, and 1 copies on QuantStudio™5 Real-Time PCR System were 23.68±0.09 and 23.76±0.06, 26.65±0.65 and 27.19±0.41, 30.16±1.05 and 30.22±0.57, 32.79±0.39 and 32.62±0.21, and 35.18±0.67 and 35.19±1.27, respectively. Similar to N1 and N2, the Ct values of RP due to Standard and Fast PCR amplification of 10000, 1000, 100, 10, and 1 copies on QuantStudio™5 Real-Time PCR System were 23.59±0.12 and 23.65±0.11, 26.08±0.62 and 26.37±0.29, 29.52±0.39 and 29.57±0.29, 32.49±0.24 and 32.55±0.63, 35.53±0.57 and 32.94±0.72. The findings indicate that almost identical Ct values were generated due to standard and Fast PCR onQuantStudio™5 Real-Time PCR System across dilution series of copy numbers of N1 target.

**Table 2:** Ct values of standard and Fast PCR-based amplification products namely N1, N2 of SARS-COV-2 & synthetic version of human RNase P, an internal control.

Target	Copy No.	QuantStudio™ 5 Real-Time PCR System		Applied Biosystems™ 7500 Fast Dx Real-Time PCR System		CFX96 Touch Real-Time PCR detection System	
		Standard	Fast	Standard	Fast	Standard	Fast
N1	10000	23.62	23.58	23.73	23.64	22.16	22.78
		±0.07	±0.13	±0.08	±0.12	±0.14	±0.03
	1000	26.23	26.58	26.25	26.57	25.36	25.79
		±0.64	±0.26	±0.57	±0.03	±0.50	±0.11
	100	29.52	28.96	29.19	29.73	27.37	28.23
		±0.82	±0.52	±0.38	±0.39	±0.14	±0.17
	10	31.96	31.28	33.29	32.81	30.62	31.83
		±0.25	±0.40	±0.19	±0.54	±0.20	±0.17
	1	35.32	35.35	36.07	36.95	34.93	34.82
		±0.24	±0.58	±0.46	±1.39	±0.05	±0.14
N2	10000	23.68	23.76	23.50	23.74	22.25	22.93
		±0.09	±0.06	±0.06	±0.11	±0.05	±0.06
	1000	26.65	27.19	26.41	26.85	25.16	25.63
		±0.65	±0.41	±0.07	±0.82	0.16	±0.49
	100	30.16	30.22	29.86	30.22	27.41	28.45
		±1.05	±0.57	±0.60	±1.35	±0.10	±0.26
	10	32.79	32.62	33.54	33.66	30.86	31.25
		±0.39	±0.21	±0.26	±1.04	±0.33	±0.19
	1	35.18	35.19	35.66	35.39	34.75	35.25
		±0.67	±1.27	±0.45	±1.11	±0.09	±0.20
RP	10000	23.59	23.65	23.61	23.73	23.22	23.86
		±0.12	±0.11	±0.04	±0.04	±0.10	±0.08
	1000	26.08	26.37	26.13	26.79	25.51	26.30
		±0.62	±0.29	±0.72	±0.86	±0.32	±0.06
	100	29.52	29.57	29.99	29.95	29.16	29.92
		±0.39	±0.29	±0.65	±1.26	±0.14	±0.29
	10	32.49	32.55	32.79	32.56	30.83	32.89
		±0.24	±0.63	±0.45	±0.82	±0.57	±0.06
	1	35.53	35.94	36.27	36.59	35.12	35.21
		±0.57	±0.72	±0.38	±0.06	±0.11	±0.10

The Ct values due to PCR amplification of starting copies- 10000, 1000, 100, 10

And 01 of N1, N2 and RP positive controls on each thermal cycler system are shown as mean ± SD of three independent experiments.

Similar to QuantStudio™5 Real-Time PCR System, Applied Biosystems™ 7500 Fast Dx Real-Time PCR System and CFX96 Touch Real-Time PCR detection Systems could generate almost identical Ct values for the same copy number of N1, N2, and RP across dilution series. Even Standard and Fast PCR amplification with the lowest copy number (1 copy/μL or 4 copies/reaction) could generate very closely-related Ct values.

Next, to confirm whether PCR efficiency remained in the acceptable limit (R^2^ value>90%) due to Fast PCR amplification, a comparison of PCR efficiency in terms of R^2^ values was made between Standard and Fast PCR. Irrespective of thermal cycler systems, all the R^2^ values generated through Fast PCR amplification were almost identical or very close to R^2^ values generated through Fast PCR amplification (Table 3), indicating that PCR efficiency was not prone to change as a result of Fast amplification.

**Table 3:** Comparison of efficiency between standard PCR and Fast PCR.

Thermal cyclers	Targets	Standard PCR (R^2^)	Fast PCR (R^2^)
QuantStudio™ 5 Real-Time PCR System	N1	0.9982	0.9819
	N2	0.9951	0.9501
	RP	0.9983	0.9859
Applied Biosystems™ 7500 Fast Dx Real-Time PCR System	N1	0.9936	0.9801
	N2	0.9939	0.9897
	RP	0.9967	0.9959
CFX96 Touch Real-Time PCR detection System	N1	0.9941	0.9971
	N2	0.9902	0.9933
	RP	0.9843	0.9949

To investigate PCR amplification efficiency, linear regression analysis was performed using Ct values generated due to PCR amplification of 10-fold serially diluted solutions containing N1, N2 and RP positive controls having 10000, 1000, 100, 10 and 01 copies each. Finally, R^2^ values were obtained.

### Performance evaluation of Fast real time PCR approach

Performance evaluation was performed in terms of (1) sensitivity, (2) specificity, and (3) precision.

### Sensitivity, specificity, and cross-reactivity of the Fast PCR approach

Irrespective of Standard or Fast PCR, all the60 Sansure kit-detected COVID-19 positive samples came out as positive when the tests were performed using RealDetect COVID-19 RT-PCR kit on QuantStudio™5 platform, AB 7500 Fast Dx Real-Time PCR System, and CFX96 Touch Real-Time PCR detection Systems, indicating a sensitivity of 100% (Table 4). Similarly, all pre-detected COVID-19 negative samples (n = 60) were detected as negative upon re-testing by both standard and Fast PCR on all thermal cycler systems, indicating 100% specificity. Together, these findings suggest that real time Fast PCR amplification could retain 100% sensitivity and 100% specificity.

**Table 4:** Performance evaluation of fast PCR in terms of sensitivity, specificity, and cross-reactivity.

Thermal cyclers	Sensitivity (%)		Specificity (%)		Cross-reactivity		
	Standard	Fast	Standard	Fast	SARS-CoV positive control	MERS-CoV positive control	SARS-CoV-2 Positive Control
QuantStudio™5 Real-Time PCR System	100	100	100	100	No amplification	No amplification	N1 and N2 amplified
Applied Biosystems™ 7500 Fast Dx Real-Time PCR System	100	100	100	100	No amplification	No amplification	N1 and N2 amplified
CFX96 Touch Real-Time PCR detection System	100	100	100	100	No amplification	No amplification	N1 and N2 amplified

For sensitivity assays, 60 Sansure kit-selected COVID-19 positive samples and 60 negative samples were re-tested by RealDetect^TM^ COVID-19 RT- PCR detection kit using the current approach of Standard and Fast PCR. For the specificity study, 60 pre-detected COVID-19 negative samples were analyzed. To check the cross-reactivity, SARS-CoV-2 specific primer pairs with SARS-CoV and MERS-CoV positive control were determined by both Standard and Fast PCR.

On the other hand, we could not detect any cross-reactivity of SARS-CoV-2 N1 and N2 sequence-specific primers pairs with SARS-CoV and MERS-CoV positive controls, although there were as usual amplifications of SARS-CoV-2 targets with these primer pairs, indicating that the primers were specific for SARS CoV-2 N gene only but not for N gene of SARS-CoV and MERS-CoV.

### Performance evaluation of Fast PCR approach as per precision analysis in terms of Ct values

Finally, we performed precision analysis by comparing the Ct values of the targets due to standard PCR and Fast PCR amplification. Table 5 shows that the medians were comparable and close to each other for standard and Fast PCR, e.g., 23.43 vs. 21.76 for N1, 22.25 vs. 21.29 for N2, and 25.21 vs. 24.96 for RP on QuantStudio™5 platform. The differences in Ct values between Standard and Fast PCR for the mentioned Ct values were 0.67, 0.96, and 0.25 for N1, N2, and RP. Similar to QuantStudio™5, ABI 7500 Fast Dx and CFX96 Touch Real-Time detection Systems could generate closely-related Ct values (medians) for standard and Fast PCR, meaning the two approaches were identical as they did not produce significant results (p>0.5). Furthermore, when mean and median were compared between Standard and Fast PCR, we observed almost similar Ct values for each thermal cycler and target. Thus, comparison of a median value with corresponding mean again demonstrate that the two approaches, namely Standard PCR and Fast PCR were identical.

**Table 5:** Precision analysis of fast PCR amplification approach in terms of Ct values.

Targets	Protocol	QuantStudio™ 5 Real-Time PCR System Standard/ Fast		Applied Biosystems™ 7500 Fast Dx Real-Time PCR System Standard/ Fast		CFX96 Touch Real-Time PCR detection System Standard/ Fast		Significance
		Median	Mean ± SD	Median	Mean ± SD	Median	Mean ± SD	
N1	Standard	23.43	22.48±5.16	16.55	17.52±2.82	16.45	16.81±2.88	NS
	Fast	21.76	21.71±5.15	16.01	17.3±4.039	16.48	17.33±2.94	
N2	Standard	22.25	22.23±5.34	17.17	17.97±2.82	16.62	17.31±2.73	NS
	Fast	21.29	21.22±6.16	15.85	17.15±4.09	16.48	17.29±3.13	
RP	Standard	25.21	25.3±2.4	27.26	27.36±3.73	23.04	22.69±4.11	NS
	Fast	24.96	24.98±3.07	25.62	26.64±6.74	21.43	21.53±3.71	

The numbers indicate Ct values of N1, N2 and RP of 60 COVID-19 positive samples and are presented as median and mean ± SD for Standard PCR and Fast PCR for the mentioned thermal cyclers. For significance level, medians of each target were compared between the Standard PCR and Fast PCR. In addition, mean and median were also compared to see whether the two PCR approaches were identical. NS indicates non-significance (P>0.05).

## Discussion

Although utmost accuracy and maximum precision of the results generated through use of any real time PCR/RT-PCR detection systems are the most important factors in a diagnostic laboratory or environmental settings, speed is another important factor to provide timely services. The world had seen how important it was to speed up sample analysis for detection of SARS CoV-2, especially during the peak time of each wave. The main aim of this study was to shorten the RT-PCR run time. The major points of consideration which had been taken into account to shorten the total run time for COVID-19 detection by RT-PCR include (1) shortening of RT time and/or reverse transcriptase inactivation time, (2) annealing time and post-annealing extension time through ramp rate manipulation. Overall, the fast PCR approach presented here may offer a reliable alternative to regular real time PCR amplification approach which would aid in shortening total PCR run time in SARS-CoV-2 detection or the approach could be modified for detection of other real time detection of pathogens during emergency and time-sensitive situations.

The study demonstrates Fast PCR amplification strategies on 3 different real time PCR systems; namely QuantStudio™5, ABI 7500 Fast Dx and CFX96 Touch Real-Time systems using a previously reported highly sensitive in-house master mix for detection SARS CoV-2 [9]. Although QuantStudio™5 and CFX96 Touch Real-Time systems are not designated Fast PCR thermal cyclers, they possess some user options to shorten RT-PCR run time, especially through manipulation of ramp rate. On the other hand, Applied Biosystems™ 7500 Fast Dx can be used as both Fast PCR and standard PCR cycler. The study demonstrates that sensitivity, specificity, and Ct values of COVID-19 samples as well as fluorescence intensity curve patterns, Cts, and R^2^ values for dilution panels of SARS CoV-2 positive controls are not compromised due to Fast PCR. demonstrated that the global means of Ct values of all samples generated in 10 laboratories by Light cycler or ABI showed results without statistical significance and these findings are in consistent with our Ct results of Fast PCR and Standard PCR for the same thermal cycler [10]. On the other hand, demonstrated that Fast real-time qPCR was prone to lose sensitivity and produce variable results [11]. There might be several reasons to explain the findings of Hilscher which were not consistent with our findings, importantly (1) Hilscher et al. used Syber Green-based master mix, whereas TaqMan-probe-based master mix was used in the present study. Syber-Green has bad reputation for generation of primer dimer, whereas TaqMan probe-based PCR does not generate primer dimer. Also, it is almost 17/18 years that Hilscher et al. performed the mentioned experiments and by this time life cycle companies have been marketing the improved version of their real time thermal cyclers. All the real time thermal cyclers which were used in the present study were recently updated versions. In the same article, Hilscher et al. reported that Fast real time PCR was not prone to lose the specificity and this part of their findings is consistent with our results, showing 100% specificity for both Standard PCR and Fast PCR amplification. Our precision analysis showed small differences in Ct values (<1) between Fast PCR and Standard PCR which are unquestionably acceptable.

Development of first commercial thermal cycler for Polymerase Chain Reaction (PCR) in 1987 [12] and discovery of thermostable DNA Polymerase in 1988 [13] have revolutionized molecular biology through analysis of nucleic acids, and these two scientific events were essential for the development and upgrading of today’s thermal cyclers and supersensitive DNA polymerase. Further additions include new fluorophores, quenchers, and supersensitive master mixes. Our in-house master mix which we used in this study and previous study was highly compatible for Fast PCR.

To our surprise, the Fast PCR run times were almost similar for ABI 7500 Fast Dx and QuantStudio™5 platforms: 48 min & 49 min, respectively. This might be due to the reason that both these machines use peltier-controlled thermal blocks. Conversely, CFX96 Touch Real-Time Detection System uses optical technology-based reduced-mass thermal blocks. Fast PCR amplification will be cost-effective and time-saving compared to conventional approach. Life science technology companies have been competing to fill the market with portable versions of PCR machines. Now there are Palm PCR, MIC PCR etc., which might be suitable for settings in schools, space, agricultural fields, jungles and other environments. MIC PCR weighing only 2 kilograms has attracted a great deal of recent attraction for environmental settings. Even, as many as 10 MIC can be operated at the same time by connecting them to one PC using Bluetooth technology.

Although rapid screening could be a good alternative to real time RT-PCR given its quick turnaround time in the range of 10 to 30 min, the strategy may fail due to its lower level of sensitivity and specificity [14]. Under the circumstances, Fast real time qPCR/RT-qPCR could be the best strategy to fight the epidemic or pandemic of concern. For example, Mic can finish PCR amplification in 39 min. This is how Fast real time PCR/RT-PCR could be preferable to rapid screening.

The present study established real time Fast PCR amplification protocols for only two conventional real time thermal cycler systems, namely QuantStudio^TM^5 (Applied Biosystems) and CFX96 Touch Real Time Detection (Bio-Rad). To use other conventional real time thermal cyclers for Fast PCR amplification, it is necessary to optimize Fast PCR protocol for each thermal cycler.

Literature searches failed to find any reports on such technical proposals regarding the Fast PCR except some technical notes from life science companies. Thus, the study may have tremendous significance to promote the use of Fast PCR amplification locally and globally.

The limitations of the study include (1) relatively small number of samples for validation and (2) rather than sample volume, we could consider concentration of sample RNAs (ng/μL) for precision analysis.

### Conclusion

As a cost-effective and time-saving approach, Fast PCR amplification using designated or conventional thermal cyclers should be preferable to standard PCR.
